# Effect of Training Healthcare Providers in Helping Babies Breathe Program on Neonatal Mortality Rates

**DOI:** 10.3389/fped.2022.872694

**Published:** 2022-05-18

**Authors:** Maria M. Mayer, Nomvuyo Xhinti, Lolly Mashao, Zolile Mlisana, Luzuko Bobotyana, Casey Lowman, Janna Patterson, Jeffrey M. Perlman, Sithembiso Velaphi

**Affiliations:** ^1^Department of Paediatrics, Nelson Mandela Academic Hospital, Walter Sisulu University, Mthatha, South Africa; ^2^Division of Education and Training, Helping Babies Breathe Programme, Resuscitation Council of Southern Africa, Johannesburg, South Africa; ^3^Department of Paediatrics, Mthatha Regional Hospital, Walter Sisulu University, Mthatha, South Africa; ^4^Department of Global Child Health and Life Support, American Academy of Pediatrics, Itasca, IL, United States; ^5^Division of Newborn Medicine, Weil-Cornell University, New York, NY, United States; ^6^Department of Paediatrics, Chris Hani Baragwanath Academic Hospital, School of Clinical Medicine, Faculty of Health Sciences, University of the Witwatersrand, Johannesburg, South Africa

**Keywords:** newborn, mortality rates, Helping Babies Breathe (HBB), simulation, training

## Abstract

**Background:**

Training in the Helping Babies Breathe (HBB) Program has been associated with a reduction in early neonatal mortality rate (ENMR), the neonatal mortality rate (NMR), and fresh stillbirth rate (FSBR) in low- and middle-income countries (LMICs). This program was implemented in five different healthcare facilities in the Oliver Reginald Tambo (ORT) District, South Africa from September 2015 to December 2020.

**Objective:**

To determine and compare the FSBR, ENMR, and NMR between 2015 before initiation of the program (baseline) and subsequent years up to 2020 following the implementation of facility-based training of HBB in five hospitals in ORT District.

**Methods:**

Records of perinatal statistics from January 2015 to December 2020 were reviewed to calculate FSBR, ENMR, and NMR. Data were collected from the five healthcare facilities which included two district hospitals (Hospital A&B), two regional hospitals (Hospital C&D), and one tertiary hospital (Hospital E). Comparisons were made between pre- (2015) and post- (2016–2020) HBB implementation periods. Differences in changes over time were also assessed using linear regression analysis.

**Results:**

There were 19,275 births in 2015, increasing to 22,192 in 2020 with the majority (55.3%) of births occurring in regional hospitals. There were significant reductions in ENMR (OR−0.78, 95% CI 0.70–0.87) and NMR (OR−0.81, 95% CI 0.73–0.90), but not in FSBR, in the five hospitals combined when comparing the two time periods. Significant reduction was also noted in trends over time in ENMR (*r*^2^ = 0.45, *p* = 0.001) and NMR (*r*^2^ = 0.23, *p* = 0.026), but not in FSBR (*r*^2^ = 0.0, *p* = 0.984) with all hospitals combined. In looking at individual hospitals, Hospital A (*r*^2^ = 0.61, *p* < 0.001) and Hospital E (*r*^2^ = 0.19, *p* = 0.048) showed a significant reduction in ENMR over time, but there were no significant changes in all mortality rates for Hospitals B, C, and D, and for the district or regional hospitals combined.

**Conclusion:**

There was an overall reduction of 22% and 19% in ENMR and NMR, respectively, from pre- to post-HBB implementation periods, although there were variations from year to year over the 5-year period and, across hospitals. These differences suggest that there were other factors that affected the perinatal/neonatal outcomes in the hospital sites in addition to the implementation of training in HBB.

## Introduction

Globally, neonatal deaths constitute about 47% of deaths in children younger than 5 years (1). The majority of these deaths occur in low- and middle-income countries (LMICs). In sub-Saharan Africa, neonatal deaths account for 37% of under-5 deaths, with a neonatal mortality rate (NMR) of 27.9 per 1,000 live births compared to a NMR of <5/1,000 live births in high-income countries (2). Globally, about a quarter (23%) of neonatal deaths are related to intrapartum hypoxia (3). An important factor associated with high NMR in LMICs is a shortage of healthcare providers (HCP) skilled in the provision of neonatal resuscitation (4–6). This is highly relevant since the training of HCP in neonatal resuscitation in facilities has been shown to significantly reduce intrapartum-related deaths by as much as 30% (7). Basic resuscitation focusing on stimulation and bag mask ventilation without the use of endotracheal intubation and/or drugs is adequate for most newborn babies at birth (7, 8).

A basic neonatal resuscitation simulation program known as Helping Babies Breathe (HBB) was developed and launched by the American Academy of Pediatrics in 2010 (9). This program teaches HCP immediate care of the newborn and basic neonatal resuscitation using simplified protocols. It focuses on HCP supporting neonates to achieve spontaneous breathing or providing bag mask ventilation within the first minute after birth (Golden Minute) for those not breathing on their own. Several studies have reported that training HCP in HBB has resulted in a reduction in fresh stillbirth rate (FSBR), 24 h, and 7-day or early neonatal mortality rate (ENMR) (10–15). Few studies have reported on the 28-day neonatal mortality rate and they have reported that training in HBB did not affect NMR (10, 14).

Several hospitals in the Oliver Reginald Tambo (ORT) district of the Eastern Cape Province of South Africa escalated the implementation of the HBB program in 2016 and continued training in this program up to 2020 to improve neonatal outcomes. In this report, we assessed the effect of training HCP in HBB on indicators of facility-based neonatal outcomes which included fresh stillbirth, early neonatal mortality, and 28-day neonatal mortality rates.

## Methods

### Study Design and Setting

**Figure 1:**
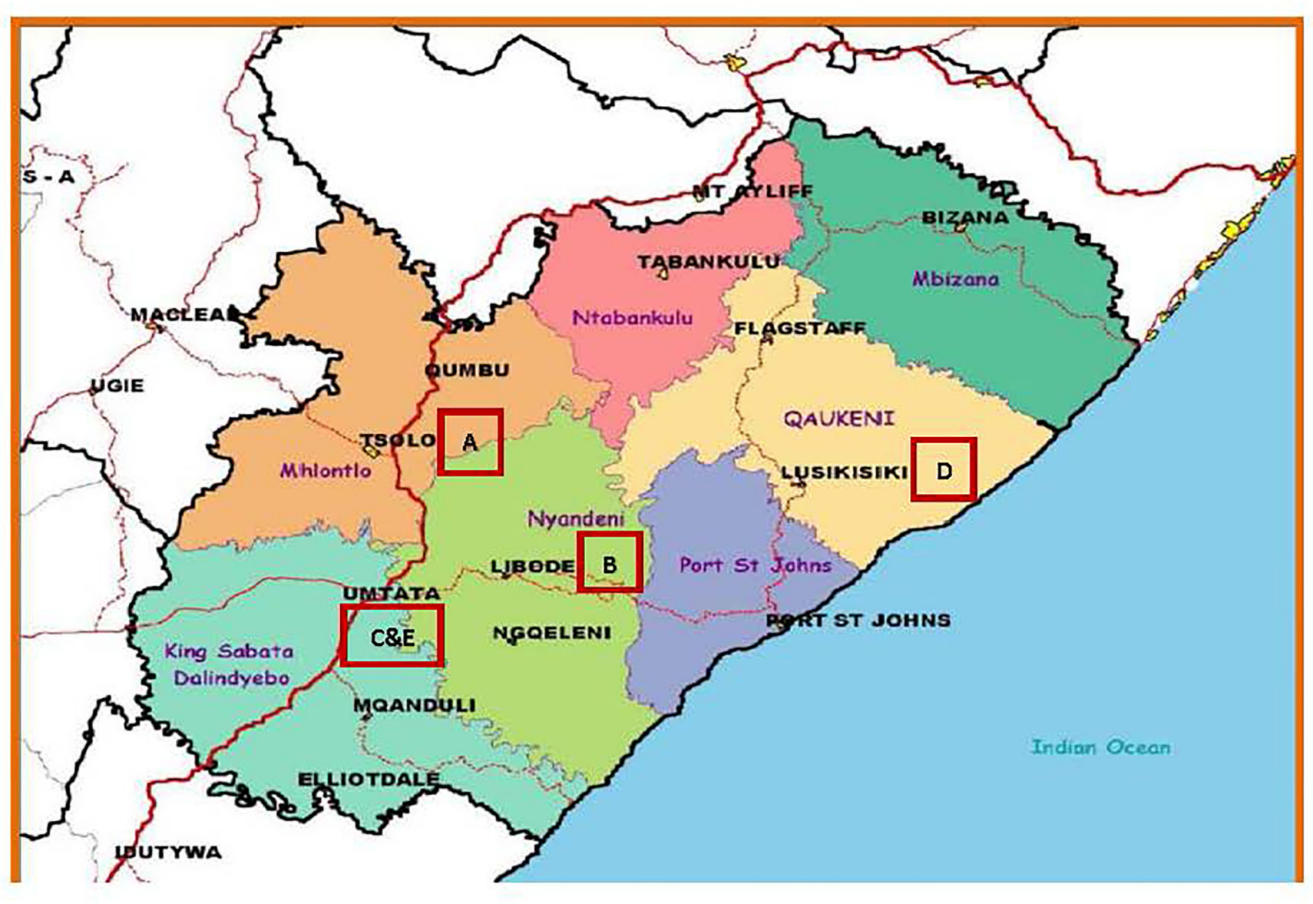
Location of different hospitals labeled as alphabets A, B, C, D, and E in the red squares or rectangles in the Oliver Tambo District in the Eastern Cape in South Africa.

This was a retrospective, observational study. The study was conducted at 5 healthcare facilities, two district hospitals (Hospitals A and B), two regional hospitals (Hospitals C and D), and one tertiary hospital (Hospital E) in the ORT district, Eastern Cape Province, South Africa (Figure 1). In the South African context, hospitals are grouped as district, regional, provincial tertiary, and central or tertiary hospitals. A district hospital receives referrals from and provides generalist support to clinics and community health centers with health treatment administered by general healthcare practitioners or primary healthcare nurses, a regional hospital receives referrals from and provides specialist support to a district hospital and where healthcare users require the expertise of teams led by resident specialists and a tertiary hospital offers similar services to regional hospital but in addition, it offers subspecialty services and has a responsibility of training HCP. The ORT district has a population of approximately 1.5 million people and covers an area of 12,141 square km, and is predominantly a rural region (16). The tertiary hospital (Hospital E) is the referral center for all hospitals in the ORT district and hospitals within the borders of districts neighboring ORT. It is the main hospital offering neonatal critical care, and the only hospital providing specialized services including neonatal surgery and subspecialty services. Management of high-risk neonates occurs in regional and tertiary hospitals and it includes offering nasal continuous positive airway pressure (nCPAP) only for neonates weighing ≥ 800 g and offering invasive mechanical ventilation (IMV) only to those weighing ≥ 1,000 grams at birth. Regional hospital C offered nCPAP and IMV to a limited extent and Hospital D offered only nCPAP, but not IMV. Regional hospital D did not have an obstetrician during the study period. All facilities capture all births as they happen daily, including stillbirths, in a birth register. Outcomes of live births are recorded in the labor and delivery rooms as well as in the neonatal wards. Neonates are nursed in the neonatal wards/areas until discharged. Post-discharge, sick neonates who need admission are admitted to neonatal wards and if they demise, their information is captured under neonatal deaths. Most of the deaths during the neonatal period in the province occur within facilities. Therefore, neonates who were not admitted post-discharge were assumed to have survived. HBB was implemented, using the training of trainers model for all hospitals, and hospitals C, D, and E had facility-based mentoring, following the training. The mentors were midwives with advanced midwifery training. Trained HCP were encouraged to continue training other HCPs at their own hospitals. A full-day HBB training covering all scenarios was conducted annually in all hospitals. In addition, hospitals C, D, and E had onsite mentors on a full-time basis and hospitals A and B were visited by a mentor from hospitals C and E every 3 months or when needed. There were 4,795 HCP trained over these 5 years and 16.7% (*n* = 802) were trained as facilitators.

### Data Analysis

Total births, stillbirths, and neonatal deaths and their time of occurrence in the five facilities were counted and were expressed as rates for all hospitals combined and for individual hospitals. FSBR was defined as number of neonates weighing 500 grams and above, born with no signs of life and with no degenerative body changes (maceration) as determined by the attending HCP at delivery per 1,000 total births. ENMR was defined as deaths of neonates born with signs of life and death within the first 7 days after birth per 1,000 live births. NMR was defined as the deaths of neonates born with signs of life and death within the first 28 days of life per 1,000 live births. Comparisons for the different indicators (FSBR, ENMR, and NMR) were made between the pre- (2015) and post- (2016–2020) HBB intervention period, using odds ratios (OR) with 95% CIs. We assessed if the changes in the indicators improved with time throughout the intervention period using linear regression analysis. Locally weighted scatterplot smoothing (LOWESS) was used in regression analysis to create a smooth line through the time plot, in order to see the relationship between time periods and mortality rates. Differences or changes were considered to be significant if they had *p* values < 0.05. This study was approved by the Walter Sisulu University Human Research Ethics Committee (Protocol number 064/15).

## Results

There were 123,898 total births conducted in the five hospitals over the study period. Annual total births increased from 19,275 in 2015 to 22,192 in 2020 (Table 1). 55 percent of births were conducted in the regional hospitals, 26% in the district hospitals, and 19% in the tertiary hospital. Overall, in comparing the pre- (2015) and post- (2016–2020) HBB implementation periods there was no significant change in the FSBR (OR−0.93, 95% CI 0.79–1.09, *p* = 0.363), while there was a significant reduction in ENMR (OR−0.78, 95% CI 0.70–0.87, *p* < 0.001) and NMR (OR−0.81, CI 95% 0.73–0.90, *p* < 0.001) (Table 2).

**Table 1:** Annual number of births in the different hospitals.

**Year**	**District hospitals (Hospitals A&B)**	**Regional hospitals (Hospital C&D)**	**Tertiary hospital (Hospital E)**	**All hospitals**
	***n* (%)**	***n* (%)**	***n* (%)**	***N* (%)**
2015	4,992 (25.9)	10,524 (54.6)	3,759 (19.5)	19,275 (100)
2016	4,748 (24.7)	10,387 (54.1)	4,080 (21.2)	19,215 (100)
2017	5,155 (26.1)	11,036 (55.8)	3,587 (18.1)	19,778 (100)
2018	5,525 (26.0)	11,705 (55.1)	3,998 (18.8)	21,228 (100)
2019	5,757 (25.9)	12,346 (55.6)	4,107 (18.5)	22,210 (100)
2020	5,705 (25.7)	12,470 (56.2)	4,017 (18.1)	22,192 (100)
Total	31,882 (25.7)	68,468 (55.3)	23,548 (19.0)	1,23,898 (100)

**Table 2:** Mortality rates and odds ratios comparing before and after implementation of Helping Babies Breathe program.

	**2015**	**2016**	**2017**	**2018**	**2019**	**2020**	**Comparing 2015 and 2016–2020**
Indicators	Rates, per 1,000 total births for stillbirths and per 1,000 live births for neonatal mortality rates						OR (95% CI)
Fresh stillbirth rate	9.44	9.47	10.01	9.14	7.02	8.47	0.93 (0.79–1.09), *p* = 0.363
Early neonatal mortality rate (7 day)	20.78	17.39	16.87	16.44	16.62	14.40	0.78 (0.70–0.87), *p* < 0.001
Neonatal mortality rates (28 day)	24.48	20.44	20.10	20.13	20.55	18.57	0.81 (0.73–0.90), *p* < 0.001

**Figure 2:**
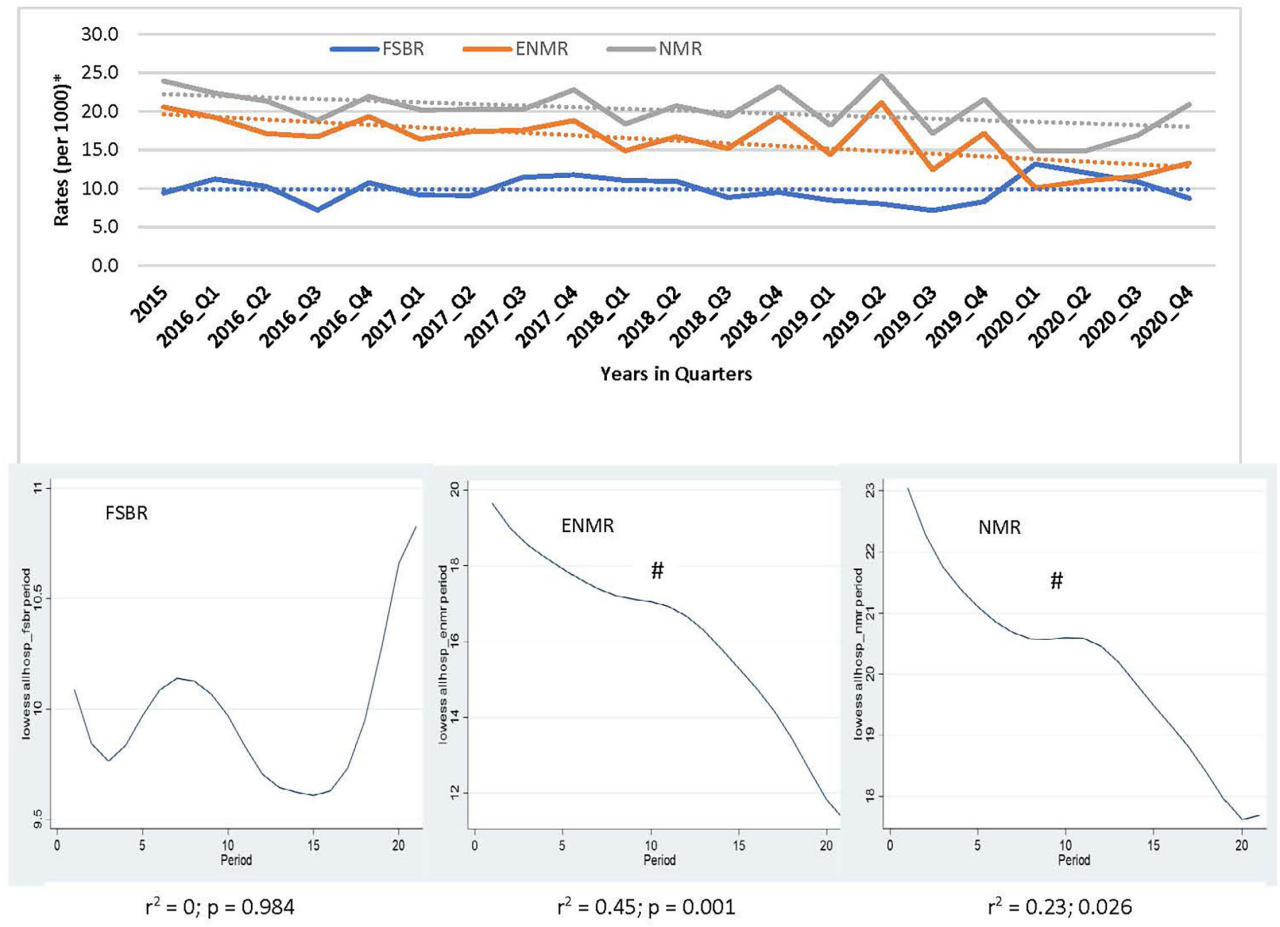
Trends in fresh stillbirth rate (FSBR), early neonatal mortality rate (ENMR), and neonatal mortality rate (NMR), with locally weighted scatterplot smoothing (LOWESS) regression analysis in the lower panel of the Figure for all hospitals combined (Hospitals A to E). ^#^Statistically significant reduction (*p* < 0.05). ^*^Per 1,000 total births for fresh stillbirth rates (FSBR) and per 1,000 live births for early neonatal mortality rate (ENMR) and neonatal mortality rate (NMR).

**Figure 3:**
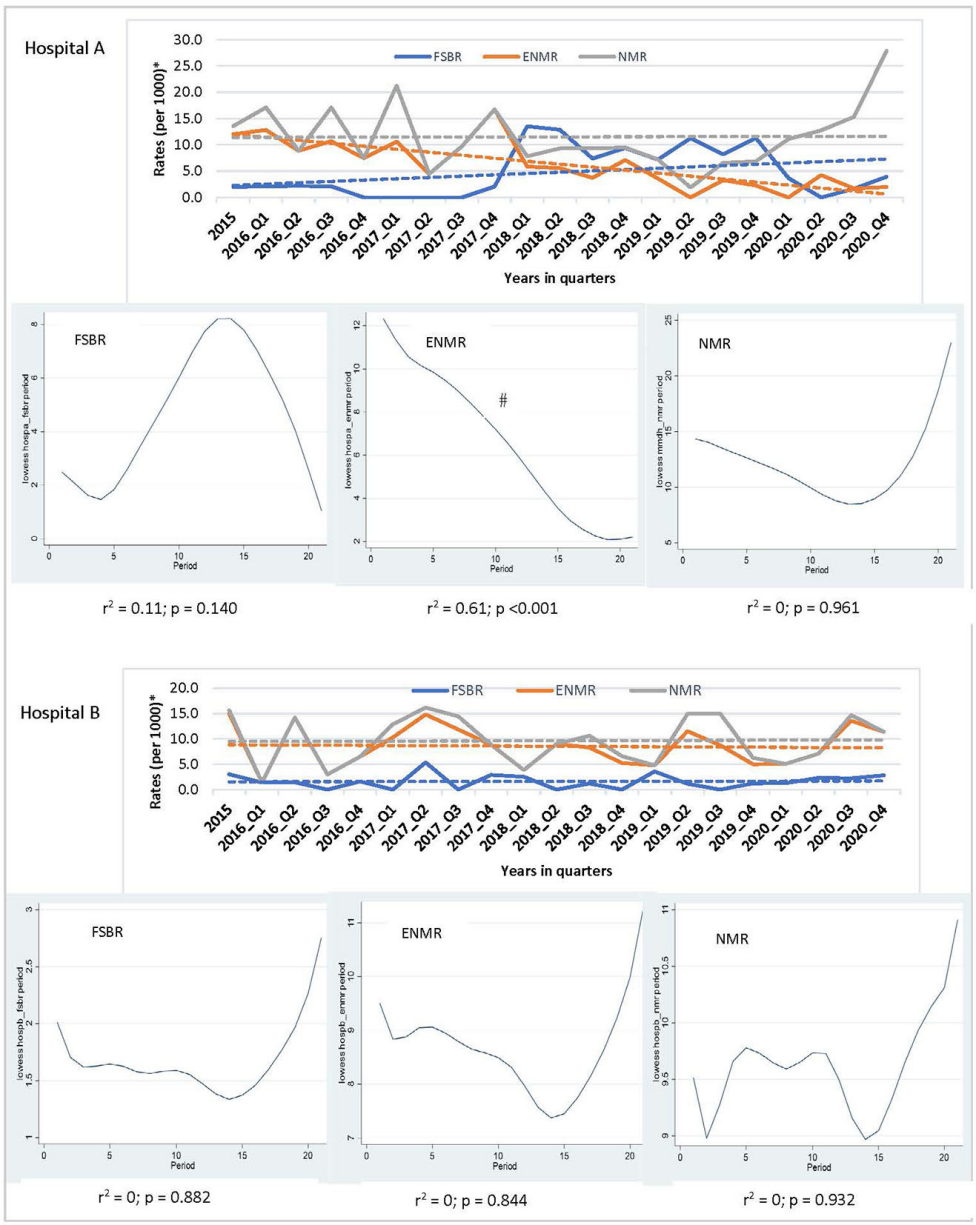
Trends in fresh stillbirth rate (FSBR), early neonatal mortality rate (ENMR), and neonatal mortality rate (NMR), with locally weighted scatterplot smoothing (LOWESS) regression analysis in the lower panel of the Figure for regional hospitals (Hospitals A and B). ^#^Statistically significant reduction (*p* < 0.05). ^*^Per 1,000 total births for fresh stillbirth rates (FSBR) and per 1,000 live births for early neonatal mortality rate (ENMR) and neonatal mortality rate (NMR).

**Figure 4:**
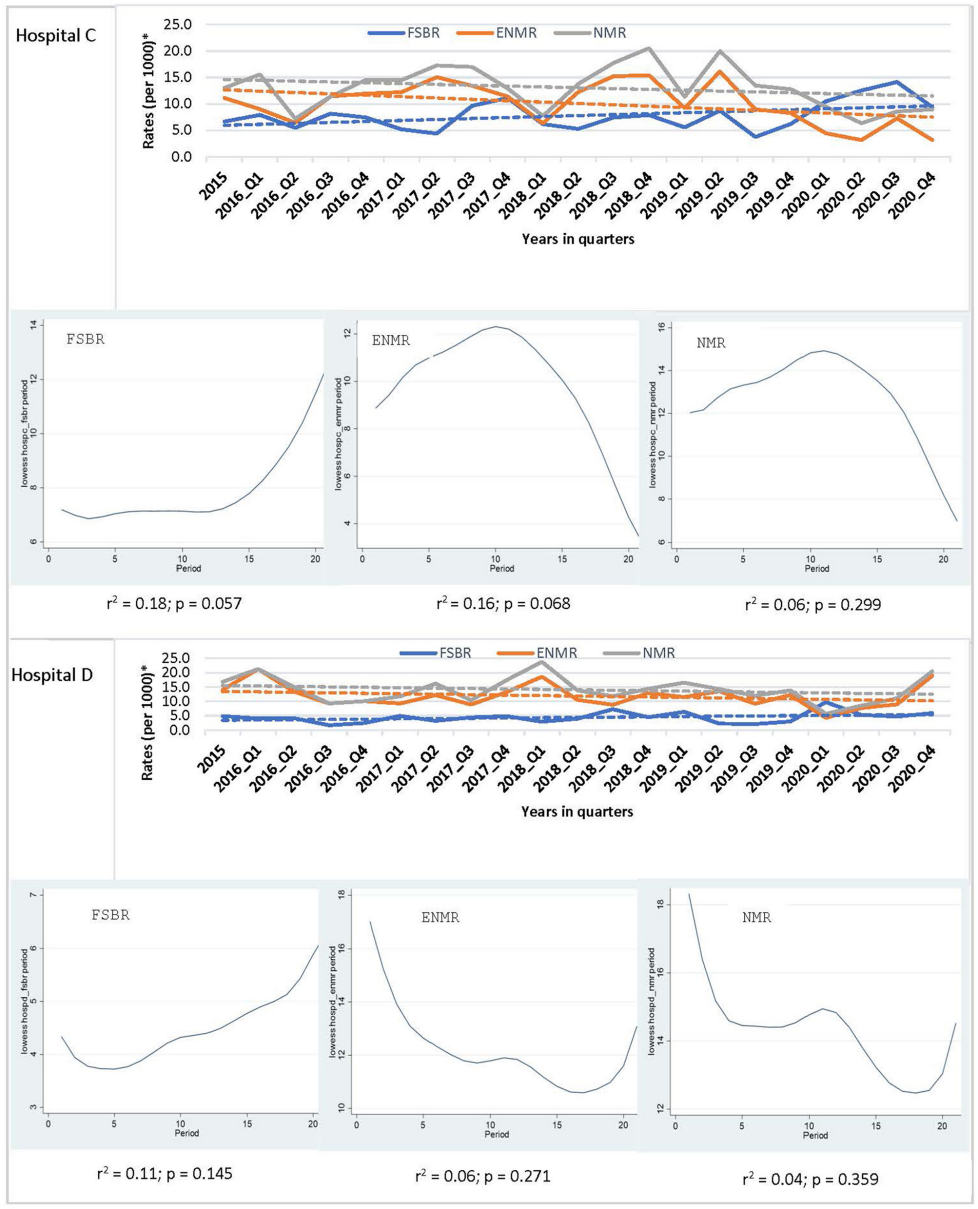
Trends in fresh stillbirth rate (FSBR), early neonatal mortality rate (ENMR), and neonatal mortality rate (NMR), with locally weighted scatterplot smoothing (LOWESS) regression analysis in the lower panel of the Figure for regional hospitals (Hospitals C and D). ^*^Per 1,000 total births for fresh stillbirth rates (FSBR) and per 1,000 live births for early neonatal mortality rate (ENMR) and neonatal mortality rate (NMR).

**Figure 5:**
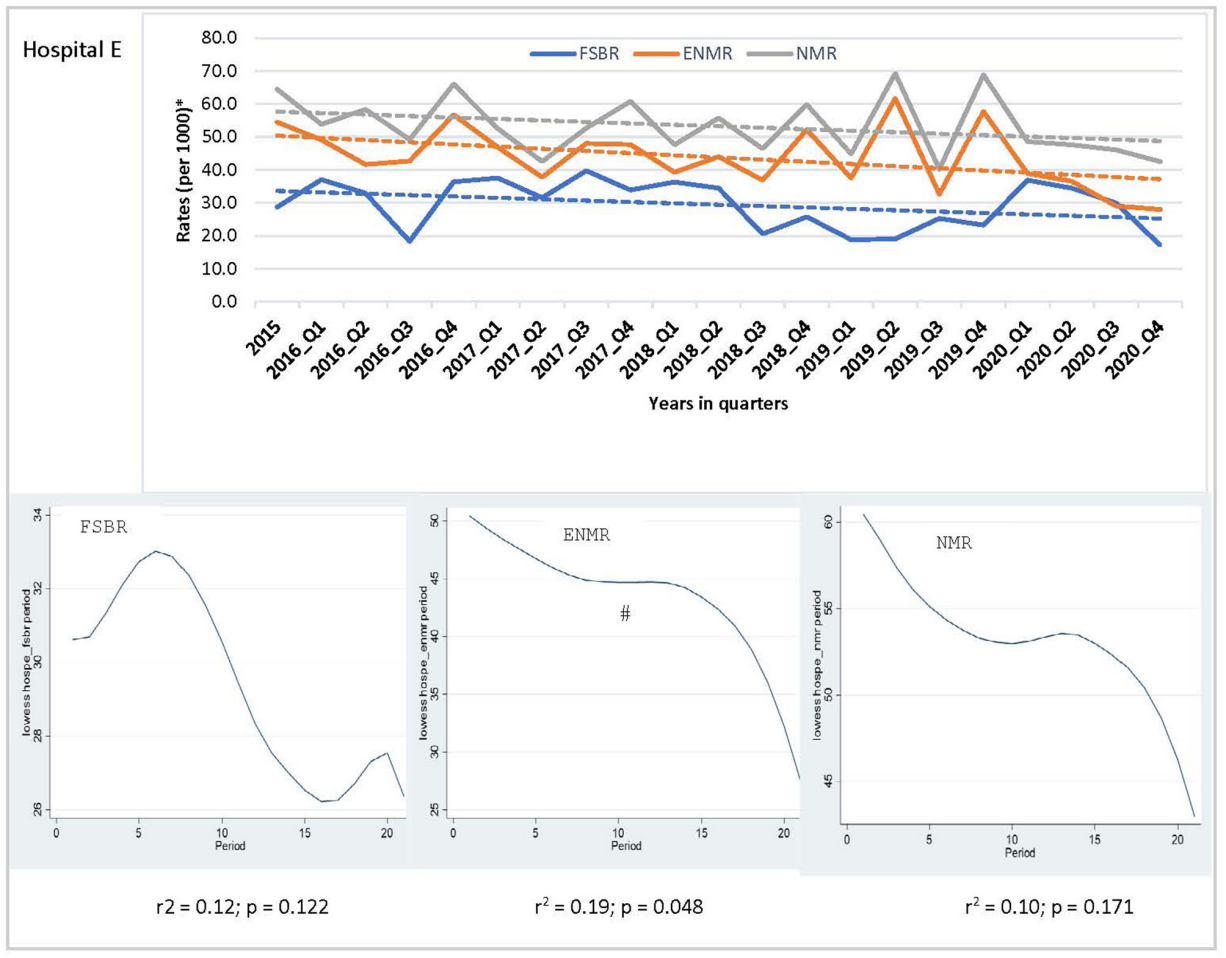
Trends in fresh stillbirth rate (FSBR), early neonatal mortality rate (ENMR), and neonatal mortality rate (NMR), with locally weighted scatterplot smoothing (LOWESS) regression analysis in the lower panel of the Figure for tertiary hospital (Hospital E). ^#^Statistically significant reduction (*p* < 0.05). ^*^Per 1,000 total births for fresh stillbirth rates (FSBR) and per 1,000 live births for early neonatal mortality rate (ENMR) and neonatal mortality rate (NMR).

In examining the trends over the five-year period for hospitals combined, there was a linear trend in reduction in ENMR (*r*^2^ = 0.45, *p* = 0.001) and NMR (*r*^2^ = 0.23, *p* = 0.026), but no significant change in the trend for FSBR (*r*^2^ = 0.0, *p* = 0.984) with time on the implementation of HBB (Figure 2). The ENMR decreased from about 20 to under 12 per 1,000 live births, and similarly, the NMR decreased from 23 to just below 18 per 1,000 live births from 2015 to the last quarter of 2020. In looking at the individual hospitals, there was a significant reduction in trends of ENMR from 12 to 2 per 1,000 live births from 2015 to 2020 (*r*^2^ = 0.61, *p* < 0.001) only in hospital A among the district hospitals. There were no significant changes in the trends of FSBR and NMR in both district hospitals (Figure 3). There were no significant changes in trends of all mortality rates in both regional hospitals (Figure 4). There were also no significant changes in trends in mortality with the district or regional hospitals combined in all mortality rates. There was a significant reduction in ENMR in the tertiary hospital from 50 to just below 30 per 1,000 live births from 2015 to the end of 2020 (Figure 5).

## Discussion

This study has shown that implementing training in HBB in the ORT district in the Eastern Cape, South Africa, was associated with a 22% reduction in ENMR and a 19% reduction in NMR, when comparing the pre- (2015) and post- (2016–2020) HBB implementation periods, but no effect on FSBR. In assessing the trends over the five-year period for the hospitals combined, there was strong evidence that training in HBB was associated with a linear reduction in ENMR and NMR over time, but not in FSBR. However, when examining the individual hospitals this effect was not the same in all hospitals. Three hospitals (two regional and one district hospital) did not show a linear reduction in ENMR, while one district hospital and the tertiary hospital showed a significant reduction in ENMR.

The finding that the effect of training in HBB showed a linear trend in reduction in ENMR but not in NMR suggests that its impact is greater during the early neonatal period, highlighting the importance of continuity in neonatal care, providing a full package of neonatal care protocols instead of focusing on a specific time point. In comparing the pre-and post-intervention periods, the effect of training in HBB on ENMR is similar to that observed in other low-resource settings (17). It further emphasizes the importance of training HCP in basic neonatal resuscitation using simulation. There was no significant change in the FSBR before and after HBB implementation which is a different finding from what has been reported previously from other LMICs (10, 11, 13). This difference is most likely related to differences in approach to monitoring fetuses during labor and/or managing neonates who are born with no signs of life at birth. In a setting where there is good intrapartum monitoring and stillbirths are diagnosed in-utero than intrapartum, and/or if there is a high threshold to call every neonate who is not breathing a fresh stillborn, effect of training in HBB might not be seen on FSBR. Whereas, in settings where every baby who is born not breathing is considered a neonate born with apnea, training in HBB is most likely to have an impact on FSBR. In this study, we were not able to differentiate whether the FSB was diagnosed antepartum or intrapartum, therefore, unable to explain the lack of effect of training in HBB on FSBR as was seen in other studies. Findings of no change in NMR in some hospitals with training in HBB are similar to that reported in other studies (10, 14). The lack of effect of training in HBB in NMR is most likely due to training in resuscitation having an impact on intrapartum hypoxia-related deaths but not on deaths related to prematurity or infections during the neonatal period. The other reason for this lack of impact could be due to inadequate resources including lack of equipment, limited availability of neonatal retrieval services, and understaffing.

This study reflects the positive effect of a dedicated simulation clinical training program in immediate neonatal care (18). The overall trend in reduction in most indicators, though not statistically significant for all indicators, is encouraging in that it reflects the potential that HBB training has in reducing fresh stillbirths and neonatal deaths in all facilities in the ORT district, as has been reported in other LMIC (10, 11, 19). The effect of this training on different indicators varied from one hospital to another. This suggests that there are other factors that played a role in changes in the mortality rates other than training in HBB or that HBB training on its own was not adequate. Regarding training in HBB, the differences could be due to the role played by local trainers in providing sustained training and mentoring to other HCP, and the high attrition rate of trainers and trained HCP as movement and relocation of staff occurs frequently in this province. The tertiary hospital with a significant reduction in ENMR may be attributed to the fact that the hospital is located within a city and therefore is less likely to have attrition of staff, and therefore the training and mentoring were consistent. Against this argument is that one of the regional hospitals is located in the same city and is within a quarter-mile of the tertiary hospital. The other reason for the tertiary hospitals having a significant improvement in ENMR is that it has more pediatric specialists including a neonatologist compared to other hospitals and has access to the equipment required for the management of high-risk infants. This highlights the importance of offering comprehensive neonatal care for one to be able to achieve a reduction in perinatal mortality rates. Conversely, in district hospitals doctors are often juniors with no experience in neonatal care and cover all the sections of the hospital and therefore are not stationed permanently in the neonatal wards, resulting in degeneration in skills gained during HBB training and a compromise in the continuity of care. The ORT district is rural with vast geographical distances between hospitals, which is often a challenge to get trainers, offering to follow-up and provide mentoring in some of the remote hospitals. It is important that HBB training in the future should utilize other technologies such as telesimulation that allows facilitators to continue training and mentoring while remote from the targeted facility. Therefore, digital platforms could reinforce training in LMIC and should be a future initiative for training in rural provinces (20). Simulation by remote facilitation or telesimulation has been shown to be feasible for neonatal resuscitation training and is associated with significant improvements in knowledge and performance (21, 22). It offers an effective alternative to conventional training in neonatal resuscitation among healthcare providers especially in those who are working in the remote areas (23).

The strength of this study is that it was pragmatic in design examining different types of hospitals with different resources. Furthermore, it looked at pre- and-post HBB implementation, but also examined trends of the different indicators over a five-year period. The limitation of this study is that there are several potential confounders that were not adjusted for, which include assessing available resources in the different hospitals and the performance of local trainers over time. The other limitation is that specific causes for perinatal/ neonatal deaths were not analyzed specifically, to identify intrapartum hypoxia-related deaths, which are the deaths that are more likely to be impacted by training in HBB. The information on characteristics of newborns, for example, incidence of prematurity and interventions available/offered; and quality of healthcare in the different hospitals were not collected or monitored. These could explain the differences among hospitals. These factors have been shown to explain differences in neonatal mortality rates across countries and regions (24–26).

## Conclusion

Training in HBB was associated with a reduction in ENMR and NMR when comparing pre- and post- program implementation. The trend was significant for a reduction in ENMR over the five-year period, but this was noted in the tertiary and district but not in the regional hospitals suggesting that training in HBB alone is not adequate for some facilities. This highlights the importance of providing comprehensive packages of perinatal/neonatal healthcare services for the whole region for all facilities to realize the reductions in perinatal/neonatal care indicators. This comprehensive care should include other educational packages focusing on obstetric and ongoing neonatal care, provide adequate and skilled nursing and medical staff, provide basic equipment for neonatal care including that which offers basic respiratory support such as continuous positive airway pressure and availability of transportation of patients from the district to tertiary hospitals.

## Data Availability Statement

The raw data supporting the conclusions of this article will be made available by the authors, without undue reservation on request.

## Ethics Statement

This study was reviewed by the Walter Sisulu University Human Ethics Research Committee. Written informed consent for participation was not required for this study in accordance with the national legislation and the institutional requirements instead verbal consent was obtained.

## Author Contributions

All authors listed have made a substantial, direct, and intellectual contribution to the work and approved it for publication.

## Funding

This work was supported in part by the American Academy of Pediatrics through a global grant from Ronald McDonald House Charities.

## Conflict of Interest

The authors declare that the research was conducted in the absence of any commercial or financial relationships that could be construed as a potential conflict of interest.

## Publisher's Note

All claims expressed in this article are solely those of the authors and do not necessarily represent those of their affiliated organizations, or those of the publisher, the editors and the reviewers. Any product that may be evaluated in this article, or claim that may be made by its manufacturer, is not guaranteed or endorsed by the publisher.
